# A Snapshot Imaging Spectrometer Based on Uniformly Distributed-Slit Array (UDA)

**DOI:** 10.3390/s22093206

**Published:** 2022-04-21

**Authors:** Yan Xu, Chunlai Li, Shijie Liu, Guoliang Tang, Jianan Xie, Jianyu Wang

**Affiliations:** 1 Hangzhou Institute for Advanced Study, University of Chinese Academy of Sciences, Hangzhou 310024, China; xuyan@shanghaitech.edu.cn (Y.X.); liushijie@ucas.ac.cn (S.L.); 2 Key Laboratory of Space Active Opto-Electronics Technology, Shanghai Institute of Technical Physics, Chinese Academy of Sciences, Shanghai 200083, China; tangguoliang@mail.sitp.ac.cn (G.T.); xiejianan@sitp.ac.cn (J.X.); 3 University of Chinese Academy of Sciences, Beijing 100049, China

**Keywords:** snapshot, uniformly distributed-slit array, spectral reconstruction, video spectral imaging

## Abstract

Herein, we propose a system for a snapshot video hyperspectral imaging method based on a uniformly distributed-slit array (UDA) coding plate that not only effectively improves the scanning speed of spectrometers but also achieves a high spectral fidelity of snapshot videos. A mathematical model and optical link simulation of the new system are established. The analysis results show that the proposed method can more efficiently collect information and restore the spectral data cube, and the spectral smile of the system is less than 4.86 μm. The results of the spectral performance and external imaging tests of the system show that the system has the ability to collect spatial spectrum video information with a frame rate of 10 Hz and identify dynamic targets, laying a foundation for the design of a system with a higher frame rate and resolution.

## 1. Introduction

Spectral imaging technology is one of the research hotspots of modern remote sensing and is applied to agriculture [1,2], mineral exploration [3], and other fields. The purpose of an imaging spectrometer is to obtain the spatial and spectral information of the target, and there are three types of data acquisition: time scanning, spatial scanning, and snapshot. The first two of these can be classified as scanning. Time scanning is limited by the bandwidth of the band-pass filter, resulting in errors in reconstructing the image, while spatial scanning reduces the recognizability of spectral imaging due to changes in the external environment or the movement of the target itself. Scanning imaging spectrometers have the disadvantage that dynamic target information cannot be fully obtained in motion scenarios such as large airport aircraft take-off and marine vessel formation cruising activities.

Conventional hyperspectral imaging systems mostly use a single slit scanning method to acquire image spectral information. The acquisition of a surface field of view target spectral data cube by a snapshot imaging spectrometer can be completed in the integration time of a single shutter. A snapshot imaging spectrometer takes less time to acquire 3D data than a scanned imaging spectrometer and effectively suppresses both types of errors caused by conventional methods.

With the continuous development of detector arrays [4,5], large format 2D focal plane arrays are gradually meeting researchers’ needs for data acquisition accuracy. Conventional imaging spectrometers are mostly based on the use of scanning methods to collect information; these take more time to collect data while obtaining a finer spectral resolution, and changes in the imaging targets and imaging environments in this process are a huge challenge to the inherent ability of conventional systems to collect information.

Thanks to the advent of high-resolution sensors, various snapshot imaging spectrometers [6,7,8,9,10,11] have been established. For instance, Hagen and Kudenov analyzed a variety of snapshot spectroscopic imaging techniques in 2013; Maione et al., presented a spatially heterodyned snapshot imaging spectrometer in 2016; Xie et al., introduced a snapshot imaging spectrometer based on a pixel-level filter array (PFA) in 2021, among others.

Descour and Dereniak [12,13] proposed a snapshot spectral imaging system based on computed tomography (CTIS) that can reconstruct a three-dimensional spectral cube with spatial and spectral dimensions from a set of acquired two-dimensional projections that integrate spectral signals from different scene locations on the sensor. The main advantage of CTIS [14] is that it can make the system layout very compact, but the main disadvantage is the high process requirements of the components and the high computational complexity.

Snapshot hyperspectral imaging Fourier transform (SHIFT) [15,16,17] provides a complete 3D data set in one period of integration time, eliminating the need to scan parts or filters and enabling compactness and vibration insensitivity. The main advantage of SHIFT is that it can obtain image information in real time, but the main disadvantage is that recovering a three-dimensional “space-spectrum” data cube is time consuming and computationally complex.

Coded aperture snapshot spectral imaging (CASSI) [18,19,20], based on the theory of compression perception, enables the sampling of hyperspectral cube data at each moment in a sparse, low-dimensional manner at first, followed by high-precision spectral reconstruction. The main advantages of this method are the low cost of the coding element and the high speed of spectral data acquisition, but the main disadvantage is that sparse assumption based on the natural scene increases and the reconstruction error increases, while the computational complexity also increases when the sampling rate decreases.

In spectral imaging detection, researchers expect to be able to obtain the most realistic three-dimensional spectral data cube of the most realistic target scene at the highest resolution and the fastest speed. However, without exception, they all have their own advantages and disadvantages, as well as their own suitable application scenarios. Computational imaging systems typically have faster imaging speeds than conventional imaging spectra in the information acquisition process and go further in the computational complexity of reconstructing spectral images. Computational imaging systems find it difficult to deal with real-time, full-resolution information, limiting their application in critical projects. Therefore, a snapshot imaging spectrometer with a simple structure, low computational complexity, and rapid reconstruction of spectral images has great research value.

Based on the low computational complexity of the full sampling of conventional scanning systems and the advantages of the fast imaging of CASSI systems, this paper designed a periodic uniformly distributed-slit array spectral imaging (UDACSI) system. It is equivalent to the periodic expansion of the conventional slit spectroscopy and the periodic dispersion matching of a large surface detector in the spectral range studied. It maximizes the use of a detector for effective surface elements and improves the imaging speed under the premise of meeting the spectral range and spectral resolution required by the design.

In another paper [21], we discussed the comparative study of conventional scanning systems, CASSI systems with different sampling rates, and systems proposed in this paper in terms of spectral data acquisition speed and spectral fidelity. While conventional scanning systems can achieve higher spectral resolution using large-area detectors, which also require more scanning time, our system can find a balance between the two. CASSI systems can reconstruct spectral data cubes at low sampling rates, theoretically having faster video imaging speeds than UDACSI systems, but reducing spectral fidelity while pursuing high-speed imaging. The signal-to-noise ratio of the CASSI system in the spectral aliased region is better than that of the full-sample uniformly distributed-slit array system [22], and the full-sampled UDACSI system is better than the non-fully sampled CASSI system in the spatial structure similarity and spectral similarity of spectral data recovery. Our system acquires data in the field of view of a pixel-level slit array, which maximizes full sampling with large-area detectors.

Our imaging spectroscopy system is mainly composed of a forward telescopic system, a uniformly distributed-slit array, a prism pair spectroscopic structure, and a complementary metal-oxide-semiconductor (CMOS) detector. We designed and assembled a uniformly distributed-slit array that can move with the micro-displacement motor, dividing the image surface covered by the detector into 15 dispersion cycles, ranging from 450 to 900 nm (visible and near-infrared). Theoretically, each dispersion cycle can achieve 35 (detector 1024 × 1024 pixels at a dual-cell merger) or 70 (detector 2048 × 2048 pixels) spectral channels. During the calibration phase, there is one-to-one periodic mapping between the pixel-level slits of the slit array and the pixels on the sensor. The spectral calibration of our system uses a monochromator (model iHR320) from a tungsten halide light source.

By using UDA, our simplified system structure increases the speed of full-sample data acquisition, making it superior to other snapshot imaging spectrometers due to its characteristics of being compact, lightweight, and low-cost. In addition, we conducted experiments based on the principles of this system and our optical design and carried out spectral calibration of our system and detection of dynamic targets. This system can achieve the acquisition and reconstruction of spectral data cubes at a 10 Hz frame rate. The optical flow [23] method was used to initially identify the motion changes of the target object in the spectral channel in the reconstructed spectral 3D data.

## 2. Principles

### System Model

The conventional single slit sweep spectral imaging system is based on the linear field of view as the information acquisition unit. It regards the need to achieve three-dimensional spectral data cube acquisition through platform sweep or pendulum mirror sweep. This paper designed a uniformly distributed-slit array coded spectral imaging (UDACSI) system to achieve spectral imaging of the field of view. In essence, the UDACSI system belongs to a special coding method of the CASSI system, and the two have a similar optical information transfer process. However, the difference is that CASSI uses two-dimensional random coding based on compression perception, and the UDACSI system only uses periodic coding in the dispersion dimension, without involving data compression, which simplifies the mathematical calculation model construction process and reduces the complexity of system reconstruction.

**Figure 1:**
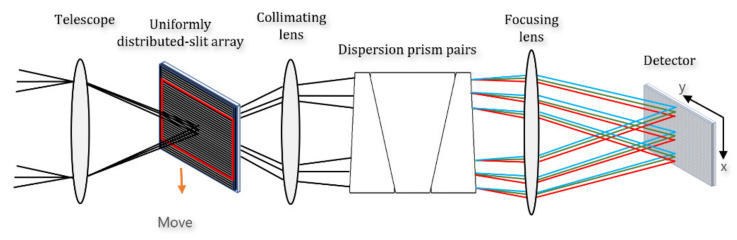
Uniformly distributed-slit array coded spectral imaging system.

Figure 1 shows a schematic of a UDACSI system, with a periodic uniformly distributed-slit array template of incident light entering the system and at the telescope’s image focal plane position, implementing spatial modulation, splitting the light by a prism, and projecting it through the lens onto the detector. A schematic diagram of the UDA at the middle of the aperture diaphragm and the upper and lower sides is shown. Spectral dispersion occurs in the *x*-axis direction; the slit array moves with the micro-displacement motor in the x-direction, and dispersion is not involved in the y-direction.

**Figure 2:**
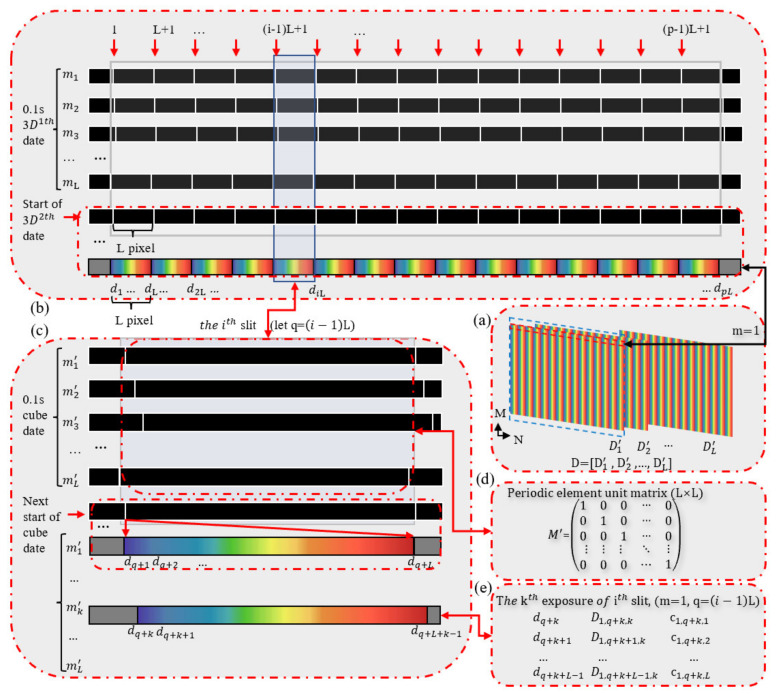
Data flow chart of the uniformly distributed-slit array coded spectral imaging system. (**a**) L times of a whole detector data acquisition during the movement of the uniformly distributed-slit array. (**b**) The L positions of the uniformly distributed-slit array corresponding to one period of detection. (**c**) The dispersion of the $i^{th}$ slit located in the aperture diaphragm. (**d**) A single slit moving within one sample period, taking the row distribution to obtain the full rank unit matrix $M^{\prime }$. (**e**) The dispersion of the $i^{th}$ slit at the $k^{th}$ exposure measurement.

Our system principle is shown in Figure 2. Considering that the system dispersion elements are one-dimensional dispersion and the direction is along the *x*-axis, it is possible to study only the dispersion direction data. Suppose an M × N × L cube of the spectral data of interest is denoted as C (Cube), where M × N is the spatial dimension, L is the spectral dimension, and $c_{m,n,l}$ (m = 1, 2, …, M; n = 1, …, N; l = 1, …, L) is the $l^{th}$ spectral channel of C at the spatial position (m, n). Suppose C is a visible white light source, and the spectrum covers the periodic dispersion range.

The detector area array is M × N pixels, the dispersion direction is along the N direction, and the number of slits in the periodic distribution is designed as P (N ≥ (P + 1) L). Corresponding to a coding mask with a size of M × PL (slit position of “1” indicates light transmission, while non-slit position of “0” indicates opacity) along the direction of the spectroscope, each pixel moved a distance of d∆, while the exposure acquisition detector two-dimensional data, moving L − 1 pixels away from the exposure L times, obtains L group data to complete the full sampling. Note the first 1/p of the uniform slit row distribution in the effective field of view corresponds to each movement and obtains $m_{1}$, $m_{2}$, …, $m_{L}$ to generate a cyclic encoding identity matrix M = ${\left[m_{1},m_{2},\ldots ,m_{L}\right]}^{T}$. Since the uniformly distributed-slit is periodic, the identity matrix M conforms to the rules of change of each slit during the movement process, as shown in Figure 2b–d. The expected spectral cube size is M × N × L of the object. The data corresponding to the uniformly distributed-slit array are the results of a single sampling cycle of the detector. The detector data are represented as D, size (M × (N + L) × L) where M × (N + L) is the two-dimensional detector face element size, L is the number of exposures required for the measurement of a single data cube, and the corresponding area array acquisition data are exposed in turn $D_{k}^{\prime }$(M × (N + L), *k* = 1, 2, …, L).

The slit array moves with the micro-displacement table, and the prism pair projects the space and spectrum of the spectral data cube, where the light of the surface field of view is located in different locations on the detector according to the principle of spatial wavelength spectroscopy. This movement process is measured L times to obtain spatial spectral data of the distribution of the detector, as shown in Figure 2a. We studied the dispersion dimension, taking *m* = 1; in Figure 2b, it can be seen that the slit of P cycle distribution in the effective field of view is simultaneously obtained in the detector spectroscopy. Our design takes into account the detector response to the wavelength, so that the spectral range of interest on the detector surface element does not produce spectral aliasing between each other. We used a micro-displacement stepper motor to control the slit array to move a pixel distance while measuring an exposure, and the movement of the slit and the data acquisition of the detector are matched. Spectral data acquisition corresponding to the spatial location of interest is completed through each slit via L exposures. We analyzed the data flow variation of a single slit over the measurement cycle, as shown in Figure 2c. We divided the slits in the effective field of view into P cycles, with a single cycle length of L, and analyzed the changes in the range of the $i^{th}$ slit ((*i* − 1) L + 1, *i*L). The initial position sequence of the slit is 1, and with the slit array moving, the slit position sequence adds 1 in turn until the slit is at the last sequence in the range. A single slit change generated by a single slit change of the 3D data is shown in Figure 2d. With the $k^{th}$ (*k* = 1, 2, …, L) second exposure, the slit position sequence is *k*, and the distribution of the spectroscopic elements on the detector is shown in Figure 2e.

When we set the $k^{th}$ measurement, there was a slit that coincided with the (*m*, *n*) pixel, and the pixel spectral data of the pixel space can be measured to form a matrix multiplication:

(1) $$\left[\begin{array}{c} D_{m,n,k}\\ D_{m,n+1,k}\\ \ldots \\ D_{m,n+L-1,k} \end{array}\right]=\left[\begin{array}{cccc} 1 & 0 & \ldots & 0\\ 0 & 1 & \ldots & 0\\ \vdots & \vdots & \ddots & \vdots \\ 0 & 0 & \ldots & 1 \end{array}\right]\left[\begin{array}{c} c_{m,n,1}\\ c_{m,n,2}\\ \ldots \\ c_{m,n,L} \end{array}\right],$$

where *n* = *(i* − 1) L + *k*, that is, the spectral data of the light of the $i^{th}$ slit in the $k^{th}$ exposure measurement.

The reconstructed spectral data cube is R with a three-dimensional size of (M × PL × L). The detector acquired spectral data [$D_{m,n,k}$, $D_{m,n+1,k}$, …, $D_{m,n+L-1,k}$], which correspond to the spectrum of R at the spatial location pixel (*m*, *n*) (*m* = 1, 2, …, M; *n* = 1, 2, …, PL).

## 3. System Design and Calibration

### 3.1. Optical System Design

**Figure 3:**
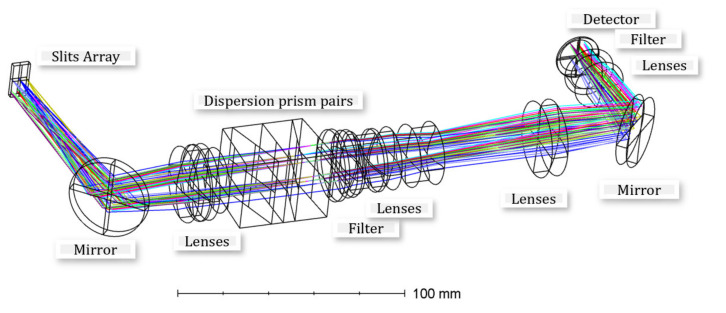
Optical system of uniformly distributed-slit array coded spectral imaging system.

The conventional single slit prism dispersion spectrometer adopts the method of single prism dispersion, and the beam of light emitted from the center and edge of the conventional slit passes through different sections of the prism, while the dispersion rate changes with the change in the top angle of the prism and the spectral dimension forms distortions. To a certain extent, this change leads to a decrease in the accuracy of the system’s spectral calibration, which makes it difficult to process and apply the imaging spectral data. Due to the characteristics of the prism dispersion itself, a single prism spectroscope has the phenomenon of spectral line bending, and in order to reduce the degree of monoprism dispersion bending, a dispersion prism pair can be used. The system samples symmetrical single prism design dispersion prism pairs and simulates our optical system using Zemax (optical design simulation software), as shown in Figure 3.

As shown in Figure 3, the coding slit is placed on the target plane of the spectrometer and the focal plane of the optical lens. The detector is placed on the focal plane of the spectrometer. We applied a set of prisms with symmetrical structures to reduce smiley faces and key effects. The band-pass filter was designed with a bandwidth of 450~900 nm, which is mainly used to reduce the influence of optical signals outside the bandwidth on the image effect.

We performed optical simulations and obtained the spectral line bending of the upper and lower edges of the field of view and the corresponding position of the middle field of view, and the spectral curvature of the dispersion prism pairs in this optical system is shown in Table 1.

By using dispersion prism pair spectroscopy, the nonlinear dispersion of different fields of view in different slits of the system is fitted and curve fitted to obtain the band of the corresponding spectral channel. Through the measurement of the spectral line bend of the dispersion prism pairs in the different fields of view of the optical system, it can be seen from Table 1 that the largest spectral bending amount is located in the long wave of 850 nm at the lower edge of the field of view, and the bending amount is 4.86 μm. Meanwhile, the cell size of the spectral channel detector CMOS is 13 μm × 13 μm, and the bending amount is less than 0.4 pixels, which has little impact on spectral calibration. In practice, the data on the edge of the detector are not used, so this bending amount meets the requirements.

### 3.2. System Structure Design

**Figure 4:**
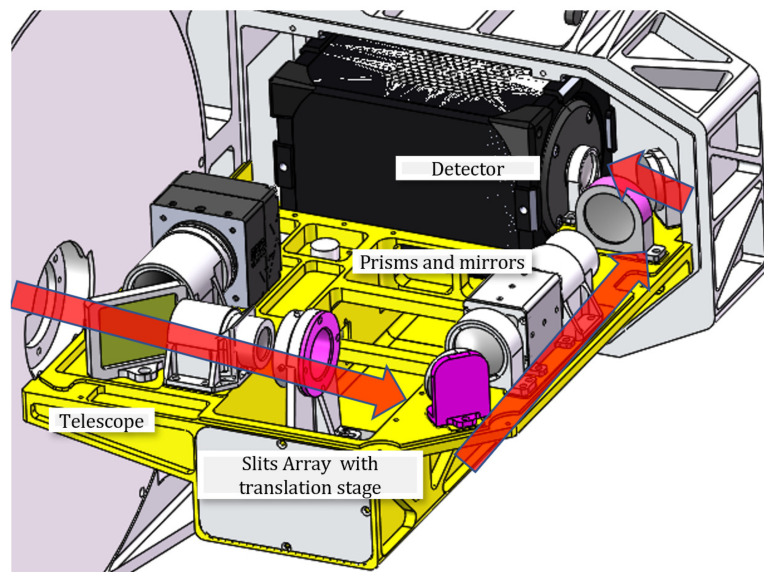
System design model.

The optical structure of the system we designed is shown in Figure 4, where the target information is aggregated through the telescope, encoded by an optical coding plate through the relay optical path, and the spectral imager achieves spectral imaging of the field of view. The optical coding plate, located at the primary focal plane of the telescope, is designed at 1024 × 1024 with pixel sizes of 13 μm × 13 μm when a dual-cell merger is selected (2 × 2 pixels). Our spectroscopic imagers with a prism and focal plane design offer compactness and high optical efficiency.

The uniformly distributed-slit array is located on the micro-displacement table, the left and right movement range is −12~12 mm, the stepper motor controls the linear motion of the position of the slit, the movement speed should match the system detector exposure frame rate, and the speed accuracy of the micro-displacement platform is high, so that the acquisition speed matches the slit array movement speed.

### 3.3. System Design 

The system includes a coaxial two-inverted telescopic, 4f structure, panchromatic imaging channel, and spectral imaging channel, wherein the spectral imaging channel adopts the aforementioned dispersion prism pair aperture coding spectroscopic technology system. The coding template was designed to meet the maximum utilization of the slit array coding board within the image detector field of view. Grating spectroscopy has a secondary or even tertiary spectrum, while prism spectroscopy does not have a multi-level spectrum. If there was a multi-level spectrum, the different wavelengths of neighboring slits in the image plane of our system would be mixed. A single prism creates more of a severe smile, and this problem can be effectively solved with three combined prisms. The prototype system was designed to verify the correctness of the principles of a uniformly distributed-slit array encoded spectral imaging system. The main technical indicators of the computing spectral imaging system are shown in Table 2 below.

**Figure 5:**
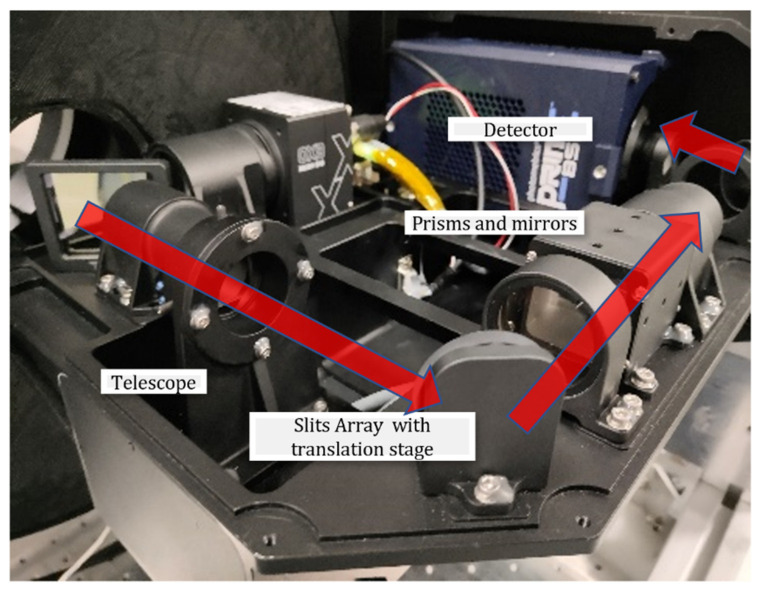
Prototype system.

As shown in Figure 5, the system is divided into two channels; one channel is a panchromatic imaging system, which adopts a high-resolution industrial camera, the charge-coupled device (CCD) camera EXO342-MU3 from SVS-Vistek in Germany; the other channel is a spectral channel, using a detector for a scientific-grade COMS camera, a high-resolution BSI Scientific CMOS of Teledyne Photometrics in Canada. This paper mainly studied the spectral channel, with the prototype realizing snapshot spectral imaging at 10 Hz and above on the basis of full sampling.

### 3.4. Spectral Resolution

**Figure 6:**
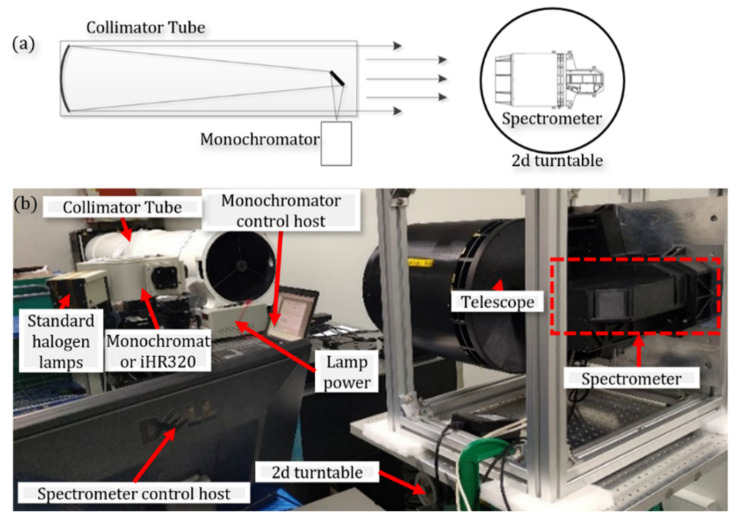
System spectral calibration schematic (**a**) and physical diagram (**b**).

**Figure 7:**
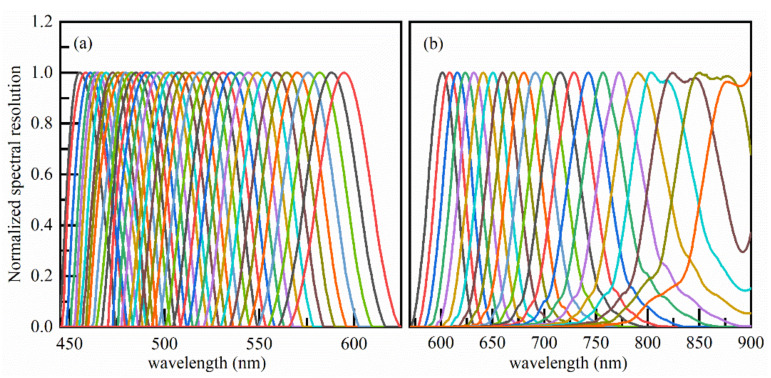
Spectral resolution test results of the hyperspectral imager. (**a**) For spectral channels 1–38 and (**b**) for spectral channels 39–60.

Spectral resolution is the most important spectral characteristic parameter of spectral imaging instruments, which determines the spectral resolution ability of the instrument. The prototype adopted the design method of the main telescope, which can realize the spectral imaging video detection in the spectral range of 450~900 nm, and a spectral calibration sampling halogen tungsten lamp of the prototype laboratory was used as the incident light source of the monochromator (model iHR320). The calibration band was 450~900 nm, the output slit width was 0.1 mm, and the step 0.5 nm was the spectral calibration for wavelength scanning, as shown in Figure 6. The spectral data collected by the prototype were processed and the normalized calibration results are shown in Figure 7 and Table 3.

The wavelength relative position accuracy of the monochromator itself was within 0.5 nm, and the statistical error when the prototype completed the spectral resolution test was within 0.2 nm. According to the calculations, after absolute position calibration was carried out by a monochromator, the absolute position accuracy of the wavelength of the final spectral calibration was at a wavelength that can be controlled at a margin of error within 0.8 nm at 550 nm.

## 4. Results

We tested the performance of external imaging tests on the test system. This included three-dimensional spectral information collection of windows for buildings and plants in a laboratory on the seventh floor. Judging from the obtained pretreatment results, it was possible to obtain visible band spectral data in the 450~900 nm band. The results show that the spectral data in the visible band can recover the spectral information of interest.

**Figure 8:**
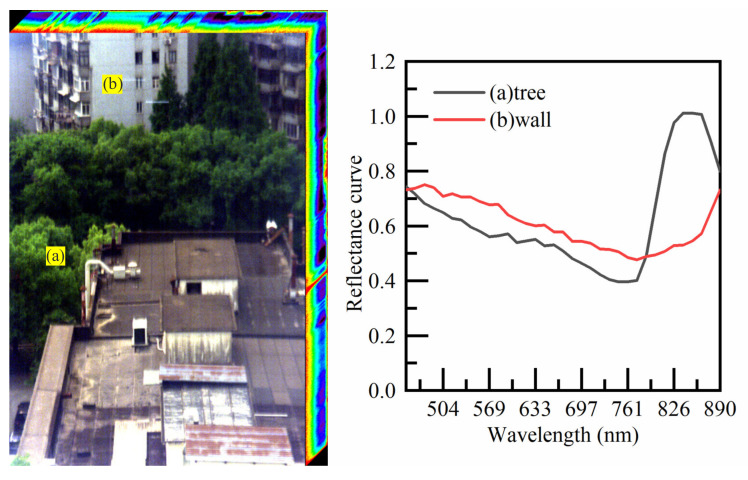
Spectral restoration image and spectral reconstruction curve.

The reconstruction of the external imaging resulted in a monochromatic image in the order of spatial position to obtain a monochromatic light image of 35 bands, as shown in Figure 8. This reconstruction clearly distinguished the spatial information of the target scene, and the spectral information of the (a) tree and (b) wall position was obtained, as shown in Figure 8, as was the spectral information of the two spatial positions and the corresponding different wavelength reflectance of the corresponding spatial positions.

The camera of the prototype system functioned at a frame rate of 350 Hz. We selected a spectral waveband of 35 for the two-pixel merge condition, with an imaging speed of 10 Hz. The fast video imaging speed leads to short integration times, low energy reception, weak signals, and poor signal-to-noise ratios. This condition means that our system is limited by energy. However, the bands of interest for the prototype system are the relatively high energy visible and near-infrared light of the solar spectrum.

**Figure 9:**
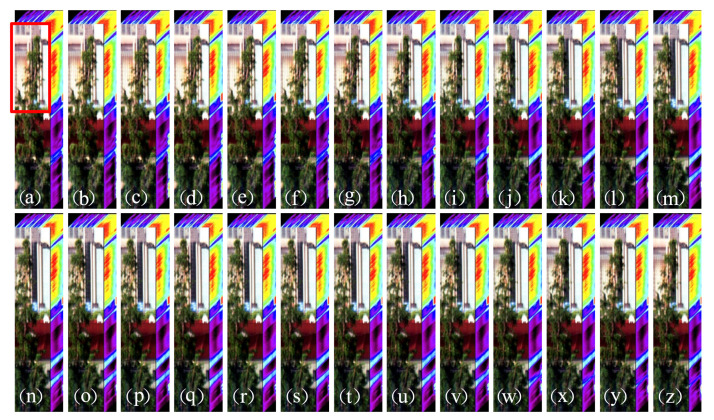
The 10 Hz frame rate for the spectral data recovery results of moving targets. (**a**–**z**) are the spectral data cubes acquired over a continuous period of 2.6 s. Reconstructed spectral data cubes are displayed by ENVI (The Environment for Visualizing Images).

Therefore, the system prototype can realize the acquisition of spectral information for dynamic targets, and the frame rate of spectral imaging can reach 10 Hz in the acquisition of spectral imaging. Figure 9 displays the results of video spectral reconstruction under the condition that the frame rate of the spectral data cube is 10 Hz. Figure 9a–z show image maps of the 26 frame spectral data cube recovered by the system in 2.6 s to the wind-blown tree movement during the time period.

Figure 9 shows the successive movements of a target dynamic tree as it swings in the scene: (a) to (m) the crown of the tree can be seen swinging to the left, (n) to (z) the crown of the tree can be seen swinging to the right. The main low-brightness dynamic trees, low-brightness walls, and high-brightness glass windows in Figure 9 can be clearly seen. Those may limit the dynamic trees that can distinguish small movements with the naked eye. We also analyzed the movement of the crown of the tree in the red box in Figure 9.

**Figure 10:**
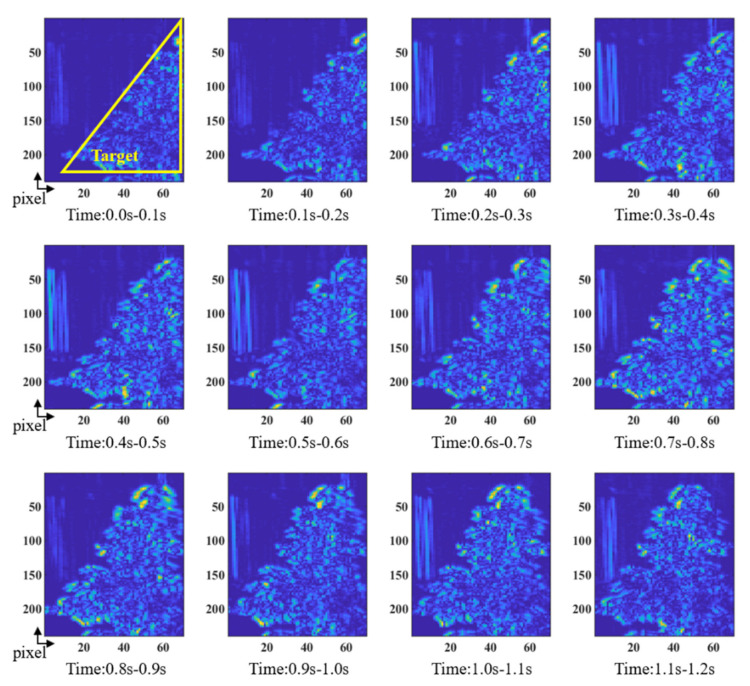
The optical flow method identifies the motion information between adjacent frames with a time difference of 0.1 s.

In this article, the Horn–Schunck optical flow method was selected to calculate the motion information of the object between the adjacent frames for each spectral channel of a continuous 13-frame spectral data cube, and a motion infographic of the first spectral channel is shown in Figure 10. The dynamic target of spectral imaging in this paper was a tree that slowly oscillates with the wind within a fixed spectrometer field of view, which meets the brightness conditions, small moving targets, and spatial consistency conditions in the optical flow method. The optical flow method uses the change of pixels in the image sequence in the time domain and the correlation between adjacent frames to find the correspondence between the previous frame and the current frame, thereby calculating the motion information of the object between adjacent frames. This motion mode utilizes the technique of edge or surface movement to detect a point of view formed between a dynamic target and the background.

Figure 10 shows the relative motion information of adjacent frames at 0.1 s, and dark blue to bright yellow indicates that the relative motion becomes larger. It can be seen that in the time from 0.0 to 1.2 s, the leaves and canopy gradually swung from the right sideways position to the middle and the leaf at the edge swung with more amplitude than in the middle.

This system can achieve the acquisition of spectral data cubes at 10 Hz and can also detect the motion information of dynamic targets in spectral channels.

The slit array of our system has 15 slits in the aperture diaphragm. When acquiring the same band and spatial size of target spectrum, our system is 15 times faster than conventional single slit scanning systems in terms of acquisition speed. Our system is not only fast in acquisition, but also simple and fast in spectral reconstruction. For example, the spectral reconstruction of our system is much simpler and faster than that of the CASSI system.

## 5. Conclusions

In this paper, a snapshot spectral imaging method based on a uniformly distributed-slit array was proposed, and a prototype system was successfully developed. The system uses the iHR320 monochromator for spectral calibration, obtaining 60 spectral channels with an average spectral resolution of better than 20 nm. The system can reconstruct the spectrum of interest and can acquire a spectral data cube every 0.1 s to achieve dynamic detection. We used the optical flow method to detect images of the same spectral channel, which can identify the motion between adjacent frames with a time difference of 0.1 s. Compared to other spectral imaging methods that sacrifice time or computational amounts, the method described in this paper meets the requirements of full sampling, low computational complexity, and hyperspectral imaging speeds. At present, the system has a signal-to-noise ratio limitation, meaning that it is difficult to apply in scenes with insufficient illumination.

## Figures and Tables

**Table 1:** Smile of optical system.

Slit Position	Wavelength	Normalized Spatial Dimensional Field of View (mm)			Smile (mm)
		0 Field	0.5 Field	1 Field	
Top edge	450 nm	−3.05374	−3.05260	−3.05018	0.00356
	550 nm	−2.86561	−2.86467	−2.86270	0.00291
	650 nm	−2.77142	−2.77047	−2.76858	0.00284
	750 nm	−2.71687	−2.71593	−2.71409	0.00278
	850 nm	−2.68176	−2.68085	−2.67909	0.00267
Center	450 nm	0.35344	0.35482	0.35694	0.00350
	550 nm	0.54427	0.54546	0.54776	0.00349
	650 nm	0.64050	0.64160	0.64400	0.00350
	750 nm	0.69618	0.69723	0.69968	0.00350
	850 nm	0.73177	0.73278	0.73527	0.00350
Bottom edge	450 nm	3.76257	3.76304	3.76552	0.00295
	550 nm	3.95619	3.95695	3.96028	0.00409
	650 nm	4.05450	4.05531	4.05894	0.00444
	750 nm	4.11136	4.11220	4.11600	0.00464
	850 nm	4.14745	4.14834	4.15231	0.00486

**Table 2:** Detailed parameters.

Parameters	Spectrometer Components	Notes
Spectral range	450~900 nm	
Spectrometer magnification	×1	
Detector cells	1024 × 1024 (13 μm × 13 μm)	2048 × 2048 (6.5 μm × 6.5 μm)
Spectral sampling	≥20 nm	
RMS radius of spot	≤6 μm	
Smile	<5 μm	
Modulation Transfer Function	>0.1	
SNR	46.9 dB	Spectral channel mean

**Table 3:** Spectral resolution data test table (medium).

Spectral Channels	Central Wavelength (nm)	Spectral Average Resolution (nm)
1~19	456.5~505.3	7.06
20~38	508.7~597.2	9.76
39~60	604.3~890.7	35.91

## Data Availability

The data presented in this study are available on request from the corresponding author.
